# Hazardous Drinking among Students over a Decade of University Policy Change: Controlled Before-and-After Evaluation

**DOI:** 10.3390/ijerph15102137

**Published:** 2018-09-28

**Authors:** Kypros Kypri, Brett Maclennan, Kimberly Cousins, Jennie Connor

**Affiliations:** 1 Department of Preventive and Social Medicine, University of Otago, Dunedin 9016, New Zealand; brett.maclennan@otago.ac.nz (B.M.); kimberly.cousins@ipru.otago.ac.nz (K.C.); jennie.connor@otago.ac.nz (J.C.); 2 School of Medicine and Public Health, University of Newcastle, Newcastle 2308, Australia

**Keywords:** college, university, drinking, alcohol, intoxication, policy

## Abstract

*Background*: Responding to high levels of alcohol-related harm among students, a New Zealand university deployed a security and liaison service, strengthened the Student Code of Conduct, increased its input on the operation of alcohol outlets near campus, and banned alcohol advertising on campus. We estimated the change in the prevalence of alcohol consumption patterns among students at the university compared with other universities. *Methods*: We conducted a controlled before-and-after study with surveys in residential colleges at the target university in 2004 and 2014, and in random samples of students at the target university and three control universities in 2005 and 2013. The primary outcome was the prevalence of recent intoxication, while we analysed drinking *per se* and drinking in selected locations to investigate mechanisms of change. *Results*: The 7-day prevalence of intoxication decreased from 45% in 2004 to 33% in 2014 (absolute difference: 12%; 95% CI: 7% to 17%) among students living in residential colleges, and from 40% in 2005 to 26% in 2013 (absolute difference: 14%; 95% CI: 8% to 20%) in the wider student body of the intervention university. The intervention effect estimate, representing the change at the intervention university adjusted for change at other universities (aOR = 1.30; 95% CI: 0.89 to 1.90), was consistent with a benefit of intervention but was not statistically significant (*p* = 0.17). *Conclusion*: In this period of alcohol policy reform, drinking to intoxication decreased substantially in the targeted student population. Policy reforms and coincidental environmental changes may each have contributed to these reductions.

## 1. Introduction

University students in Australasia, North America, and Europe have a high prevalence of hazardous drinking [1,2,3,4]. Alcohol consumption increases markedly among students upon entering university and throughout first year [5,6], while the increase among peers who do not attend university is less pronounced [6,7]. Studies have found a higher prevalence of hazardous drinking among students compared to their non-student peers [8,9], and university attendance may increase the risk of alcohol problems later in life [10,11].

Many students suffer acute harm from the intoxicating effects of their own and others’ drinking [1,2,4,12,13,14,15,16,17]. Additionally, members of the wider community living close to universities, particularly campuses with a high prevalence of hazardous drinking, are more likely to sustain damage to property, encounter vomit on walkways, witness people urinating, and be exposed to excessive noise, compared to those residing further away [18].

The University of Otago is one of eight universities in New Zealand (population 4.7 million). Located in Dunedin (population 120,000), and the majority of Otago students live in residential colleges and shared houses surrounding the campus in North Dunedin [19]. Between 2006 and 2009, in response to growing concern about disorderly behaviour [20,21,22,23,24], the university implemented several initiatives. A 2006 amendment of the *Code of Student Conduct* [25] enabled the university to reprimand and expel students for disorderly behaviour occurring on and off campus. In 2007, the University established *Campus Watch*, a security and liaison programme designed to enforce the code and support students and other residents of North Dunedin [26]. In 2009, the University banned alcohol advertising and sponsorship at events held on campus, and at university-organised events off-campus [27].

Residential colleges tightened their alcohol policies in this period [28], and the university began to monitor applications by commercial enterprises to sell alcohol in North Dunedin, objecting to licence renewals for premises that seemed to encourage excessive drinking. In 2009–2010, the University purchased two of the four large pubs next to campus [29,30], converting them into offices and a study centre. Another premises closed in 2013 [31] and re-opened in May 2016 as a ‘gastropub’ offering high-end beer and food. These pubs had been student institutions in the folklore of the university for decades [32].

The broad view within the university administration is that hazardous drinking and disorderly behaviour have decreased because of these changes [28]. An evaluation of *Campus Watch* found that drinking decreased between 2005 and 2009 among Otago students compared with students at other New Zealand (NZ) universities [33].

In this study, we examined changes in the prevalence of drinking, recent intoxication, and drinking at various locations among Otago students between 2004/2005 and 2013/2014, encompassing the period in which the aforementioned policies were introduced. This builds on our previous research [33] by including longer pre- and post-policy periods that also capture the implementation of the alcohol advertising ban. In addition, it includes surveys at residential colleges because they house a large proportion of first-year students, a group at particularly high risk of hazardous drinking [5].

## 2. Methods

NZ’s Multi-Region Ethics Committee (MEC/05/01/013) and the University of Otago Human Ethics Committee (01/117, 12/278, 14/085) approved the survey protocols.

### 2.1. Residential College Alcohol Surveys (RCAS)

We conducted a web survey of University of Otago students living in 12 Dunedin residential colleges, in April–May 2004, halfway between the beginning of the academic year and first semester exams (see http://ipru3.otago.ac.nz/demo/halls2004/). We employed many of the same questions in a web survey of the same colleges, plus two new colleges, in April–May 2014 (see http://ipru3.otago.ac.nz/demo/halls2014/). The questions relied upon for the comparison reported were worded identically in the pre- and post-study surveys.

The sampling frame was the list of residents held by the university. We sent personalised letters to all residents (2004: *n* = 2497; 2014: *n* = 3262) inviting them to participate by clicking on a personalised hyperlink sent to their university e-mail account. We sent up to one reminder letter and three reminder e-mails to residents who had not responded.

### 2.2. Tertiary Student Health Surveys (TSHS)

We conducted web surveys of randomly selected full-time intramural students aged 17–24 years, in April–May of 2005 and 2013 (see http://ipru3.otago.ac.nz/demo/) at participating NZ universities. The data collection procedures are described in detail elsewhere [2,3].

### 2.3. Measures

*Drinking patterns.* We asked RCAS participants about their drinking over the previous seven days, four weeks, and the previous calendar year (the NZ academic year runs from February to November). We classified drinkers as respondents who reported alcohol consumption during any of these periods.

We asked participants who had consumed alcohol in the previous calendar year to complete the Alcohol Use Disorders Identification Test (AUDIT) [34] in relation to that year. As the vast majority of college residents are first-year students, this typically reflected their pre-university drinking. We classified respondents scoring ≥8 on the AUDIT as having a hazardous drinking pattern upon taking up residence in the college. TSHS participants were asked about their alcohol consumption in the previous seven days, four weeks, and 12 months. We classified respondents as drinkers if they had consumed alcohol in any of these periods.

*Intoxication and locations of drinking in the previous seven days.* We asked respondents who reported drinking in the previous seven days (7-day drinkers) to complete a retrospective 7-day record for each of four location types (pub/bar/nightclub, residential college, private residence, other) in which they had consumed alcohol, indicating the number of standard drinks (10 g ethanol) and the duration of drinking in hours. We used this information to estimate a blood alcohol concentration (EBAC) for each drinking occasion [35] and designated residents with EBAC >0.08 g/dL to have become intoxicated. We assigned EBACs of zero to non-7-day drinkers.

### 2.4. Analysis

We used Stata/SE 13.1 (Stata, College Station, TX, USA) [36], adjusting the TSHS data for the sampling design, by applying weights reflecting the stratified sampling of Māori and non-Māori students. Prevalence fractions of drinking, 7-day intoxication, and drinking at various locations were compared for the 2004 and 2014 RCAS responses, and 2005 and 2013 TSHS responses. We estimated changes in 7-day intoxication prevalence separately for all respondents (i.e., including those who had not consumed alcohol in the previous seven days), and 7-day drinkers. We used logistic regression to weight estimates from the later surveys for changes between survey waves in distributions of participant gender, age, and ethnicity. We adjusted the 2014 RCAS intoxication estimates for past-year hazardous drinking to account for differences in pre-university drinking. We estimated change in the prevalence of intoxication at Otago relative to the other universities using an intervention*survey wave interaction term in a logistic regression model. We inflated standard errors to cluster respondents within universities and residential colleges, and when estimating differences in mean per-occasion consumption between RCAS waves.

## 3. Results

Of the 2497 Otago students living in the Dunedin residential colleges in 2004, 64% completed the questionnaire. A further 3% submitted responses meeting the minimum data requirement. These figures for 2014 (*n* = 3262) were 56% and 3% respectively.

Four universities (Otago, Lincoln, Victoria, and Waikato) participated in both the 2005 and the 2013 TSHS. Of the 854 Otago students invited to participate in 2005, 73% completed the questionnaire or submitted responses meeting the minimum data requirement. The response fraction in 2013 (*n* = 1228) was 61%. The combined response fractions at the other universities were 66% (*n* = 2152) in 2005 and 42% (*n* = 3301) in 2013.

Approximately two-thirds of RCAS and TSHS respondents were women (Table 1). The majority of RCAS respondents were aged 18 years, while over half of the TSHS respondents were aged ≥20 years.

Smaller proportions of men, those aged >18 years, and Asian students responded to the 2014 RCAS compared with 2004 (Table 1), while proportions of Māori, Pacific, and first-year students were larger. At Otago, there was a larger proportion of Asian respondents in the 2013 TSHS than in 2005, and a smaller proportion of European and “Other” ethnicities. At the other three universities combined, there was a smaller proportion of Asian respondents in 2013 than in 2005, and larger proportions of women, 18–19-year-olds, and students of European ethnicities.

### 3.1. Drinking Patterns

The proportion of respondents who were past-year or past-four-week drinkers did not change between 2004 and 2014 (Table 2), but the proportion who drank in the preceding seven days was smaller in 2014 than in 2004.

Hazardous drinking in the year prior to becoming a resident was also less common in 2014 than in 2004 (difference: −12.8%, 95% CI: −17.1% to −8.4%). Seven-day drinkers consumed less per drinking occasion in 2014 (mean = 7.9 standard drinks; 79 g ethanol) than in 2004 (mean = 9.7 standard drinks; 97 g ethanol), the difference attenuating slightly after adjustment for changes in respondent demographic characteristics between the surveys (difference: 1.4, 95% CI: 0.9 to 2.0).

Table 2 also shows changes in drinking status among Otago students versus students at other universities. Past-year drinking decreased at the other universities, but not at Otago. The four-week prevalence of drinking decreased overall, but non-significantly at Otago. Conversely, against a backdrop of no change at the other universities, 7-day drinking at Otago decreased, but not significantly.

### 3.2. Recent Intoxication

Table 3 summarises changes in the 7-day prevalence of intoxication, which decreased among residential college respondents and the subset of 7-day drinkers. A difference remained after adjusting for past-year hazardous drinking between the surveys (difference among all respondents: −7.4%; 95% CI: −11.8% to −3%; among 7-day drinkers only: −7.7%; 95% CI: −12.3% to −3.2%).

The 7-day prevalence of intoxication decreased between 2005 and 2013 at Otago and at the other universities combined (Table 3), and the absolute change at Otago was larger than at other universities overall and among 7-day drinkers. The difference between the change at Otago relative to the other universities among all respondents was significant (ratio of ORs: 1.46, 95% CI: 1.02 to 2.09, *p* = 0.04) but not after adjustment for changes in respondent demographic characteristics between the surveys (ratio of ORs: 1.30, 95% CI: 0.89 to 1.90, *p* = 0.17). Unadjusted (ratio of ORs: 1.34, 95% CI: 0.87 to 2.04, *p* = 0.18) and adjusted (ratio of ORs: 1.28, 95% CI: 0.82 to 1.99, *p* = 0.27) estimates among 7-day drinkers only were both non-significant.

### 3.3. Drinking Locations in the Past 7 Days

The proportion of 7-day drinkers who had consumed alcohol at on-license premises in the preceding week fell substantially from 2004 to 2014 (Table 4). The prevalence of drinking in residential colleges and ‘other’ locations remained the same, while the proportion consuming alcohol at private residences was higher in 2014 than in 2004.

There were significant decreases between 2005 and 2013 in the proportion of 7-day drinkers at Otago who had consumed alcohol at private residences and on-license premises in the previous week (Table 4). There was no change in the proportion who had consumed alcohol at a residential college, and there was a non-significant increase in the proportion who had consumed alcohol at ‘other’ locations.

A decrease between surveys in the proportion of 7-day drinkers at the other universities who consumed alcohol at private residences and on-license premises in the previous week was significant (Table 4). There was no change in the prevalence of students drinking in residential colleges and ‘other’ locations.

### 3.4. Sensitivity Analysis on Residential College Alcohol Survey Data

To investigate bias from selective non-responses, we adopted an approach previously employed to investigate non-response bias in survey data [37]. We estimated the proportion of 2004 respondents who reported drinking to intoxication among those who had participated by day 18 (i.e., the day we reached a response fraction of 59%, our final response fraction in 2014). The difference between 7-day intoxication prevalence (all respondents and 7-day drinkers only) among all respondents in 2004, versus those who responded by day 18, was <1%.

We then estimated differences in the proportion of respondents drinking to intoxication in 2004 versus 2014, based on the assumption that the true 7-day prevalence of intoxication among non-respondents was 20%, 33%, 50%, or 100% higher than among respondents. The result was differences in estimates from 2004 to 2014 by up to 1.5%.

## 4. Discussion

Our findings suggest that while the proportion of drinkers in the Otago student population did not change in the decade between the surveys, a smaller proportion of drinkers consumed alcohol or became intoxicated in the preceding 7 days. This was true even after adjusting for differences in the pre-university drinking profiles of the populations between surveys. Intoxication was also less prevalent over time among university students overall in NZ. The reduction (of around a third) was large among Otago students, but not significantly larger than it was among students at other universities. Dunedin college residents drank less per occasion in 2014 than in 2004, mainly driven by substantially decreased drinking in pubs and bars, which was only slightly offset by drinking more in their residential colleges.

In 2014, a smaller proportion of residential college students had consumed alcohol hazardously in the previous calendar year (i.e., before most became residents). This may reflect the greater proportion of 16–17-year-olds in residential colleges in 2014. While NZ law does not prohibit the consumption of alcohol at any age, people under 18 years of age cannot purchase it legally, a restriction that research suggests is effective in reducing alcohol-related harm [38]. Accordingly, this change in the demographic composition of the student population may account for some of the differences observed.

The strengths of the study include the equivalent timing of the surveys within the academic calendar, and the use of identical questions and survey methods, facilitating valid comparisons over time and between residents and wider student populations. The sensitivity analyses on RCAS-based estimates suggested that the differences in response fractions are unlikely to have substantially biased estimates of change.

The sampling design used in the TSHS meant a similar sensitivity analysis on these estimates was infeasible. Previous studies suggest the prevalence of drinking is underestimated to a greater extent when response rates are lower [37,39]. Accordingly, over-estimation of differences in intoxication prevalence between 2005 and 2013 would be less pronounced for Otago given the lesser decline in response rates there (12% versus 24% at the other universities combined), making this a conservative test of whether the interventions were associated with reduced drinking.

This and other factors may have contributed to our non-significant finding when comparing change in the prevalence of 7-day intoxication at Otago with change at the other universities. In addition to limitations of statistical power, our measure of 7-day intoxication may not have been sensitive enough to detect a modest change in drinking behaviour, for example, going from having 10 drinks in an evening to having two or three fewer drinks, or to spreading the consumption over a longer period, but without reducing their mean estimated BAC to below our threshold for classifying them as intoxicated.

We did not attempt to formally determine whether institutional policies or programmes were introduced at the other universities that could impact on student drinking. Such developments, if effective, would have biased our findings towards the null, potentially masking any effects of the Otago interventions.

While the TSHS allowed for directly comparable control sites (i.e., other universities), the RCAS included residential colleges at Otago only, limiting inferences about the Otago interventions on alcohol consumption in the colleges. It should be noted, however, that the decrease in drinking to intoxication among residents is consistent with the direction of change in the wider Otago student population, albeit of lesser magnitude. The large proportion of first year students living in the residential colleges may have different perceptions and expectations about alcohol use in the university environment than students in the wider population, many of whom are more senior. Those in their first year may be more motivated to drink to intoxication and this could be reinforced by the social aspect of college living.

During the study period, local government alcohol policies focused on the Central Business District rather than North Dunedin [40]. Legislation governing the sale of alcohol changed in 2012, but provisions with the potential to affect student drinking (e.g., Local Alcohol Policies) came into effect after the 2014 survey [41]. An exception is the blood alcohol limit for drivers under 20 years of age being reduced from 0.03 g/dL to zero, in 2011. The majority of student accommodation in Dunedin is within walking distance of campus and alcohol outlets [42], and residents do not rely heavily on private cars. Student populations at other universities, where more students rely on private cars, may have moderated their drinking to a greater extent in response to this restriction than those at Otago, potentially biasing estimates toward the null.

It is unlikely that the relative reduction in 7-day drinking and intoxication among Otago students, including those living in residential colleges, merely reflects a national trend or local government interventions. Without a randomised design, we cannot safely infer that they are due, even in part, to the strategies implemented by the university. The marked decrease in drinking at on-license premises makes the pub closures in North Dunedin a strong candidate explanation for the estimated reductions, given that drinking episodes in pubs are more likely to result in intoxication, particularly among men, than drinking in other locations [43].

Pub closures, perhaps combined with the blood alcohol limit reduction for driving, may have influenced the number of drinks Otago students consumed per occasion and where they drank. NZ research shows that the proximity of on-licensed premises to university students’ homes is associated with their drinking and related problems [44]. The reduction in the number of on-license premises in North Dunedin may have reduced market pressure for cheap drink promotions such that drinking in pubs became less appealing because of high drink prices and reduced physical accessibility requiring longer walks or taxis.

Only a small proportion of Otago students appear to have substituted drinking in pubs with drinking at other locations. There was, however, an increase in the proportion of college residents drinking in private homes in the preceding 7 days. How much these 2014 students drank at private homes compared to what their 2004 predecessors were drinking in pubs is unknown. We found that the amount consumed per occasion by residents was lower in 2014, although the overall amount consumed within residential colleges increased slightly. Focus group research conducted in this population in the early 2000s suggests that residents were drinking at their college before going to a pub [5]. Residents may now be drinking more in their college because drinking in pubs has become comparatively more expensive, and, for most, there are no practical alternative locations to continue drinking. *Campus Watch* may be reinforcing this by deterring students from drinking in public places around the campus.

Overall, there has been a shift among Otago students to drinking less than weekly and across fewer types of locations, with a large shift away from drinking in pubs. This coincided with a large reduction in the prevalence of intoxication among Otago students, consistent with what we know from previous research. Drinking in pubs, residential colleges, and private residences facilitates heavy drinking and intoxication among NZ university students relative to ‘other’ locations [43,45]. As found in that focus group research, drinking in pubs after ‘pre-loading’ at a residential college or private residence probably facilitates heavier drinking episodes.

## 5. Conclusions

The confluence of findings from surveys conducted over a decade, involving students at four NZ universities, and 12 residential colleges in North Dunedin, suggests that with concerted effort, it is possible to change a drinking culture. The university’s involvement in objecting to license renewals may have created a tipping point for businesses that had survived by breaching server laws. By issuing a clear statement of institutional values (through the *Code of Student Conduct*), introducing a policy with highly visible symbolic value (the advertising ban) and implementing a well-resourced prevention programme (*Campus Watch*), the university began attracting fewer hazardous drinkers. It seems likely that the actions of the university along with the reduction in pubs surrounding campus has helped to reduce hazardous drinking in the Otago student population.

## Figures and Tables

**Table 1:** Demographic characteristics of survey samples.

NZ Universities (TSHS)				Dunedin Residential Colleges (RCAS)				
2013		2005		2014		2004		
Other ^1^	Otago	Other ^1^	Otago	Population	Respondents	Population	Respondents	
(*n* = 1434)	(*n* = 730)	(*n* = 1356)	(*n* = 617)	(*n* = 3261)	(*n* = 1941)	(*n* = 2497)	(*n* = 1662)	
*%*	*%*	*%*	*%*	*%*	*%*	*%*	*%*	
								**Gender**
32	36	40	35	43	34	41	38	Male
68	64	60	65	57	66	59	62	Female
								**Age (years)**
1	1	2	1	9	10	3	3	16–17
21	18	18	19	69	67	62	64	18
24	20	21	21	14	14	22	20	19
53	61	60	59	7	9	13	13	20+
								**Ethnicity**
14	19	19	11	15	13	16	16	Asian
70	69	61	73	74	77	77	77	European
9	7	9	6	6	5	3	3	Māori
4	2	3	1	3	3	1	1	Pacific Islander
4	3	8	9	2	3	3	3	Other
								**Year of study**
-	-	-	-	84	82	78	78	First
-	-	-	-	11	11	13	13	Second
-	-	-	-	3	4	4	4	Third
-	-	-	-	3	3	5	5	4th year or above

^1^ Lincoln, Victoria and Waikato universities. RCAS: T Residential College Alcohol Surveys; HS: Tertiary Student Health Surveys.

**Table 2:** Changes in drinking patterns.

**Residential College Alcohol Surveys (RCAS)**	**Year**				
	***2004***	***2014***		**Change ^1^**	
	*%*	*%*	*Adj%* ^2^	*%*	*95% CI*
***All respondents***					
Prevalence of drinking	93.6	95.5	95.0	1.4	(−0.6, 3.4)
***Drinkers only*** **^3^**					
Past 4 weeks	92.0	90.4	91.1	−0.9	(−3.7, 2.1)
Past 7 days	73.9	65.8	68.7	−5.2 *	(−9.1, −1.2)
**Tertiary Student Health Surveys (TSHS)**	**Year**				
	***2005***	***2013***		**Change ^1^**	
	*%*	*%*	*Adj%* ^2^	*%*	*95% CI*
***All respondents***					
*Prevalence of drinking*					
Otago	91.7	90.1	91.0	−0.7	(−4.4, 3.0)
Lincoln, Victoria, Waikato	87.2	86.6	83.8	−3.4 *	(−6.5, −0.2)
***Drinkers only*** **^3^**					
*4-week drinking prevalence*					
Otago	95.0	90.9	92.0	−3.0	(−6.2, 0.2)
Lincoln, Victoria, Waikato	91.1	88.0	86.9	−4.2 **	(−7.3, −1.0)
*7-day drinking prevalence*					
Otago	71.2	65.3	67.2	−4.1	(−10.3, 2.2)
Lincoln, Victoria, Waikato	59.4	62.1	61.0	1.6	(−3.0, 6.3)

^1^ Difference between the 2004/2005 and adjusted 2013/2014 prevalence estimates. ^2^ Adjusted for differences in gender, age, and ethnicity of respondents between surveys. ^3^ Drinkers are defined as those who consumed alcohol in the previous four weeks and/or the previous calendar year (RCAS) or previous 12 months (TSHS). * *p* < 0.05, ** *p* <0.01.

**Table 3:** Changes in the 7-day prevalence of intoxication between surveys.

**Residential College Alcohol Surveys (RCAS)**	**Year**					
	***2004***	***2014***		**Change ^1^**		
	*%*	*%*	*Adj* ^2^	*%*	*95% CI*	*Relative change*
***Intoxicated (EBAC > 0.08) in the last 7 days***						
*Residential colleges*						
All respondents	44.9	33.4	32.7	−12.1 **	(−17.1, −7.1)	−27.0%
7-day drinkers	67.2	54.9	54.8	−12.4 **	(−17.2, −7.5)	−18.5%
**Tertiary Student Health Surveys (TSHS)**	**Year**					
	***2005***	***2013***		**Change ^1^**		
	*%*	*%*	*Adj* ^2^	*%*	*95% CI*	*Relative change*
***Intoxicated (EBAC > 0.08) in the last 7 days***						
*Otago University*						
All respondents	40.3	24.9	26.1	−14.2 **	(−20.3, −8.1)	−35.2%
7-day drinkers	60.1	41.8	42.6	−17.4 **	(−25.6, −9.2)	−29.0%
*Lincoln, Victoria, and Waikato universities*						
All respondents	24.5	19.1	18.2	−6.4 **	(−9.9, −2.8)	−26.1%
7-day drinkers	45.9	34.8	35.1	−10.8 **	(−16.8, −4.9)	−23.5%

^1^ Difference between the 2004/2005 and adjusted 2013/2014 prevalence estimates. ^2^ Adjusted for differences in gender, age, and ethnicity of respondents to the 2004/2005 and 2013/2014 surveys. * *p* < 0.05, ** *p* < 0.01.

**Table 4:** Prevalence of drinking at residential colleges, private residences, on-licence premises and ‘other’ locations among students who drank alcohol in the preceding seven days.

**Residential College Alcohol Surveys (RCAS)**	**Year**				
	***2004***	***2014***		**Change** **^1^**	
	*%*	*%*	*Adj%* ^2^	*%*	*95% CI*
***Drinking locations in the last 7 days***					
*University of Otago college residents*					
Residential college	78.9	77.5	77.3	−1.2	(−7.4, 4.3)
Private residence	26.5	32.5	33.7	7.2 *	(0.7, 13.7)
Pub	82.0	57.7	58.4	−23.6 **	(−28.8, −18.4)
Other ^3^	20.8	22.8	23.8	3.1	(−0.6, 6.7)
**Tertiary Student Health Surveys (TSHS)**	**Year**				
	***2005***	***2013***		**Change** **^1^**	
	*%*	*%*	*Adj%* ^2^	*%*	*95% CI*
***Drinking locations in the last 7 days***					
*Otago students*					
Residential college	16.8	15.9	19.4	2.6	(−2.7 to 7.9)
Private residence	77.1	70.4	67.8	−9.2 **	(−16.1 to −2.4)
Pub	73.7	51.9	53.2	−20.5 **	(−28.4 to −12.6)
Other ^3^	24.2	31.4	30.4	6.2	(−1.3 to 13.7)
*Lincoln, Victoria, Waikato students*					
Residential College	12.1	15.9	14.4	2.3	(−1.3 to 6.0)
Private residence	77.3	67.6	67.9	−9.4 **	(−14.8 to −4.1)
Pub	61.5	50.9	49.9	−11.6 **	(−17.7, −5.4)
Other ^3^	25.8	26.3	26.7	0.9	(−4.6 to 6.5)

^1^ Difference between the 2004/2005 and adjusted 2013/2014 prevalence estimates. ^2^ Adjusted for differences in gender, age, and ethnicity composition between respondents to the 2004/2005 and 2013/2014 surveys. ^3^ Other locations include restaurants, public outdoor settings, private functions, and sports clubs. * *p* < 0.05, ** *p* < 0.01.
